# Acute Arterial Thrombosis after Covered Stent Exclusion of Bleeding Mycotic Pseudoaneurysm: Treatment Using Catheter-Directed Thrombolysis

**DOI:** 10.1155/2011/264053

**Published:** 2011-04-28

**Authors:** Sarah Palestrant, M. Grace Knuttinen, Ron C. Gaba, James T. Bui, Charles A. Owens

**Affiliations:** Section of Interventional Radiology, Department of Radiology, University of Illinois at Chicago, 1740 West Taylor Street, MC 931, Chicago, IL 60612, USA

## Abstract

Conventional absolute contraindications to catheter-directed thrombolysis include active or recent hemorrhage and the presence of local vascular infection, both of which increase the risk of procedure-related complications such as bleeding and systemic sepsis. For this reason, lytic therapy of arterial thromboembolism under these circumstances is generally precluded. Herein, we describe a unique case of safe catheter-directed lysis of an acutely thrombosed iliac artery following covered stent placement for treatment of an actively bleeding infected pseudoaneurysm. Our management approach is discussed.

## 1. Introduction

Catheter-directed thrombolytic agent infusion represents an effective and well-established method of restoring blood flow in acute and subacute thromboembolic arterial occlusion [1]. This therapy is associated with a 61.9–85.7% efficacy rate and a low major hemorrhagic complication rate of approximately 5.1% [1–3]. Traditional contraindications to catheter-based lytic therapy include active or recent hemorrhage, which increases risk for procedure-related bleeding complications, and infection at the site of thrombosis, which may result in systemic sepsis [4–6]. To date, there is limited data on the safety of catheter-based thrombolysis in the presence of these conditions. Herein, we describe a unique case of safe catheter-directed thrombolysis of an acutely thrombosed iliac artery pursued immediately after covered stent insertion was performed for treatment of an actively bleeding infected pseuodoaneurysm. A discussion of our management approach and a review of the relevant literature are presented.

## 2. Case Report

Institutional review board approval is not required for single retrospective case studies at our hospital. A 46-year-old woman was referred to interventional radiology (IR) for image-guided drainage of a large pelvic abscess discovered after she presented to the emergency department (ED) with fever and leukocytosis. The patient had a history of stage IV rectal cancer (cloacogenic type) for which she underwent palliative laparoscopic diverting sigmoid colostomy 26 months prior. In separate procedures, she also underwent subsequent pelvic exenteration, abdominoperineal resection, en bloc vaginectomy, bilateral salpingo-oophorectomy, right groin lymphadenectomy, and perineal reconstruction with a rectus abdominis myocutaneous flap, as well as receiving a high external beam radiation dose to the pelvis (45 Gy) and systemic chemotherapy with 5-fluorouracil and oxaliplatin. 

**Figure 1:**
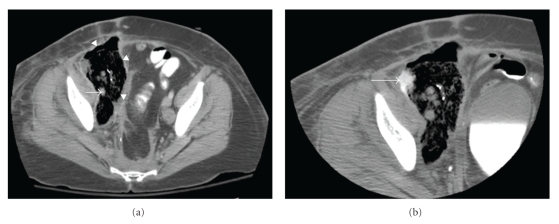
Contrast-enhanced pelvic CT (a) reveals 12 × 7 cm air- and fluid-filled abscess (arrowheads) in abdominal right lower quadrant. Encasement of right external iliac artery (arrow) by abscess noted. (b) Presence of bowel contrast (arrow) within collection confirms fistula to colon.

**Figure 2:**
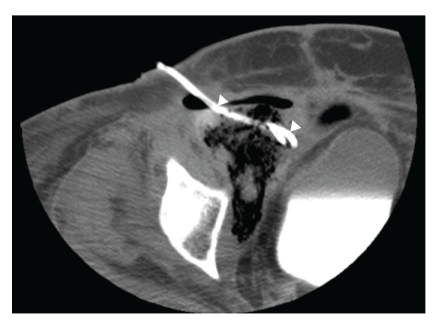
Noncontrast CT image following percutaneous drainage shows 12 French catheter (arrowheads) in anterior aspect of pelvic abscess.

Computed tomography (CT) scan obtained in the ED demonstrated a large heterogeneous air and fluid filled collection with thick irregular enhancing walls in the region of the patient's original primary tumor, suggestive of fistula formation to adjacent bowel with resultant abscess formation within a large residual necrotic pelvic tumor (Figure 1). The pelvic mass also encased the right iliac vessels, with the right external iliac artery completely encompassed by infection. Subsequent percutaneous CT-guided catheter drainage of the right pelvic abscess aspirated foul-smelling debris (Figure 2). At the time of drainage, extra-anatomic arterial bypass with concurrent iliac artery embolization was recommended to the primary surgical service given the envelopment of the iliac arteries within infected debris and the associated risk of infected pseudoaneurysm formation of the involved vessels. The patient clinically improved and was subsequently discharged from the hospital several days later with the drainage catheter left in place.

Approximately three weeks later, the patient returned to the ED with active bright-red bleeding from the right lower quadrant drain as well as from her ostomy (950 mL of blood was present in the patient's drainage bag). She was tachycardic and hypotensive, with a heart rate ranging from 140 to 150 beats per minute and a systolic blood pressure measuring approximately 50–60 mm Hg prior to vasopressor administration. Marked skin palor was evident. Laboratory assessment revealed a hemoglobin level of 8.1 g/dL. The patient was resuscitated with intravenous fluid hydration and transfusion of four units of packed red blood cells. 

**Figure 3:**
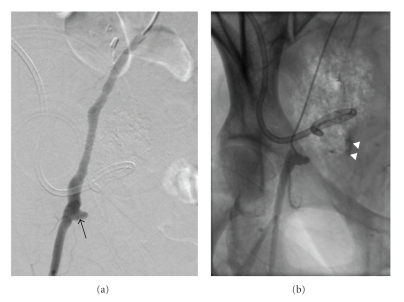
Digital subtraction (a) and unsubtracted (b) images from right iliac arteriogram reveal contrast extravasation (arrowheads) from right external iliac artery pseudoaneurysm (arrow).

Immediate concern was for rupture of an infected right iliac artery pseudoaneurysm, and, therefore, the patient was urgently transferred to the IR angiography suite for emergent preoperative angiography and possible endovascular therapy. Through a left common femoral artery 5 French 10 cm long sheath (Pinnacle; Terumo Medical Corp., Somerset NJ) a 5 French Omni Flush catheter (AngioDynamics, Queensbury NY) was utilized for bilateral pelvic arteriography followed by selective right common iliac arteriography using a 5 French angled hydrophilic catheter (Glidecath; Terumo Medical Corp., Somerset NJ). Arteriography demonstrated active hemorrhage from a pseudoaneurysm arising from the right external iliac artery segment traversing the known pelvic abscess (Figure 3). The Glidecath (Terumo) was immediately exchanged for a 7 mm balloon catheter (Ultra-Thin Diamond; Boston Scientific, Natick MA) that was advanced over a 0.035 inch stiff guide wire, positioned across the pseudoaneurysm and inflated in an attempt to tamponade further bleeding. 

After discussing the angiographic findings and potential treatment options with the colorectal and vascular surgery services, a request was made to place covered stents along the right external iliac artery to exclude the bleeding pseudoaneurysm. The patient was deemed to be an unsuitable candidate for emergent operative intervention given poor clinical status and low likelihood for postoperative survival following an open procedure. Furthermore, an operative approach from the right groin was thought to be unfavorable due to local cancer spread, radiation-related tissue fibrosis, and suspected healing impingement due to poor tissue quality, lymphadenectomy, and presence of tissue flaps. 

**Figure 4:**
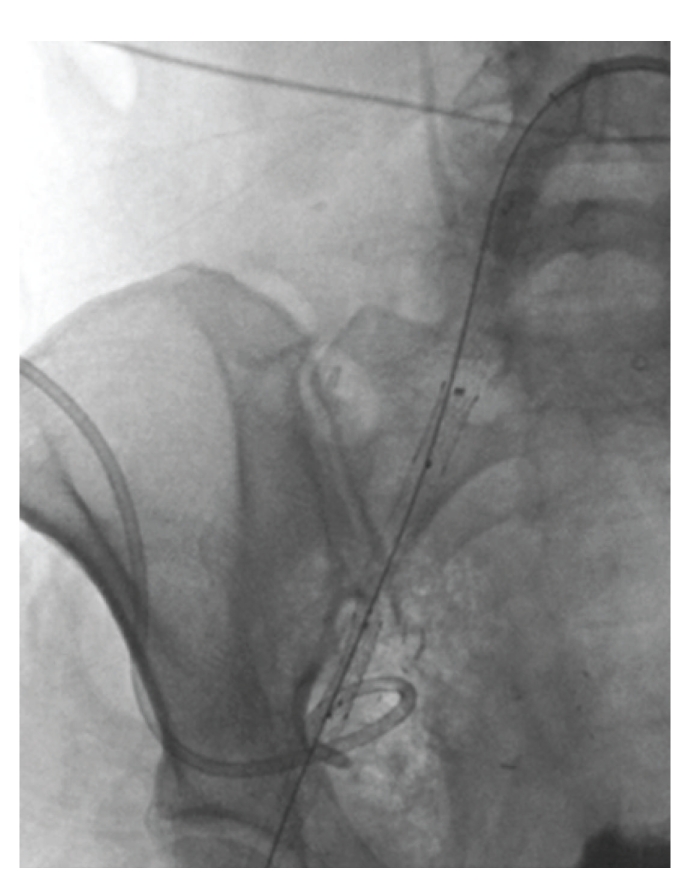
Fluoroscopic spot image demonstrates covered stents deployed across right common and external iliac arteries.

The 5 French left groin sheath was then exchanged for a 9 French 55 cm long sheath (Raabe Flexor Check-Flo; Cook Medical, Bloomington IN), which was guided into the distal right external iliac artery. Three overlapping 9 mm × 60 mm, 8 mm × 80 mm, and 8 mm × 60 mm Fluency-Plus (Bard, Murray Hill NJ) covered stents were then deployed across the right external iliac artery (Figure 4) to the level of the right femoral artery, successfully excluding the bleeding pseudoaneurysm. Three stents were used in order to cover the entire length of vessel traversing the right pelvic abscess. No heparin was administered at the time of stent deployment due to active bleeding. Due to potential risk of infectious complications, the patient was treated with intravenous piperacillin-tazobactam (Zosyn; Wyeth, Madison NJ) and metronidazole (Flagyl; Pfizer, New York NY), which were continued for the duration of her ten-day hospital stay. 

**Figure 5:**
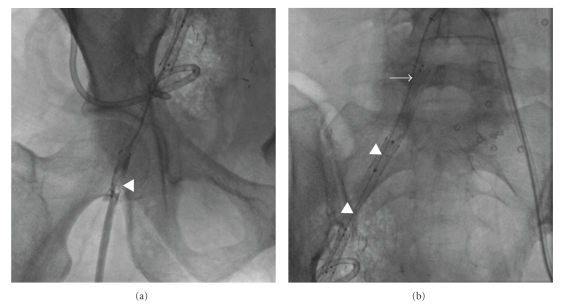
Right common femoral arteriogram (a) reveals acute thrombosis (arrow) of distal segment of stented artery. Of note, unsaved manual injections of contrast better documented thrombus burden throughout entire course of stented iliac artery segment. Fluoroscopic spot image (b) shows 5 French catheter (arrow) and infusion wire (arrowheads) system used for catheter-directed thrombolysis.

Immediate postdeployment arteriography revealed thrombosis of the newly stented right external iliac artery with thrombus extending to the right common femoral artery bifurcation (Figure 5), likely related in part to prolonged balloon tamponade. The initially asymptomatic right lower extremity gradually began to demonstrate signs and symptoms of significant limb ischemia prompting repeat discussion of surgical intervention. At this point, treatment options were discussed further with the surgical service followed by the patient's family. Interventional considerations included catheter-directed thrombolysis, mechanical thrombectomy, stent-in-stent placement, surgical embolectomy, arterial bypass, and limb amputation. A decision was made to revascularize the right lower extremity using catheter-directed low-dose alteplase thrombolysis, as this was believed to offer the best chance for vessel recanalization and limb salvage while minimizing potential morbidity based on our excellent institutional experience using low-dose alteplase infusion. Mechanical thrombectomy, while possible, was felt to carry risk of clot disruption, distal arterial embolic disease, and secondary tissue ischemia. Stent-in-stent placement was not believed to permit sufficient return of luminal caliber in the setting of large thrombus volume and was felt to be associated with risk of distal embolization. Surgical embolectomy and arterial bypass were not pursued due to the hostility of the tumor-laden and postradiation surgical field. Limb amputation, while possible, was felt to represent a salvage therapy if limb-sparing maneuvers were unsuccessful. 

For thrombolysis, a 5 French Glidecath (Terumo) was advanced into the proximal right external iliac artery, and a coaxial 0.035 inch SOS infusion guide wire (Medtronic, Minneapolis MN) was positioned within the thrombosed right common femoral artery. Alteplase (Activase; Genentech, South San Francisco CA) was delivered via the catheter and coaxial wire with a total infusion dose of 0.48 mg per hour (Figure 5), with systemic heparin titrated to maintain a partial thromboplastin time (PTT) of approximately 60–80 seconds. As per institutional routine, complete blood count, coagulation profile, and fibrinogen levels were monitored every six hours during lysis.

**Figure 6:**
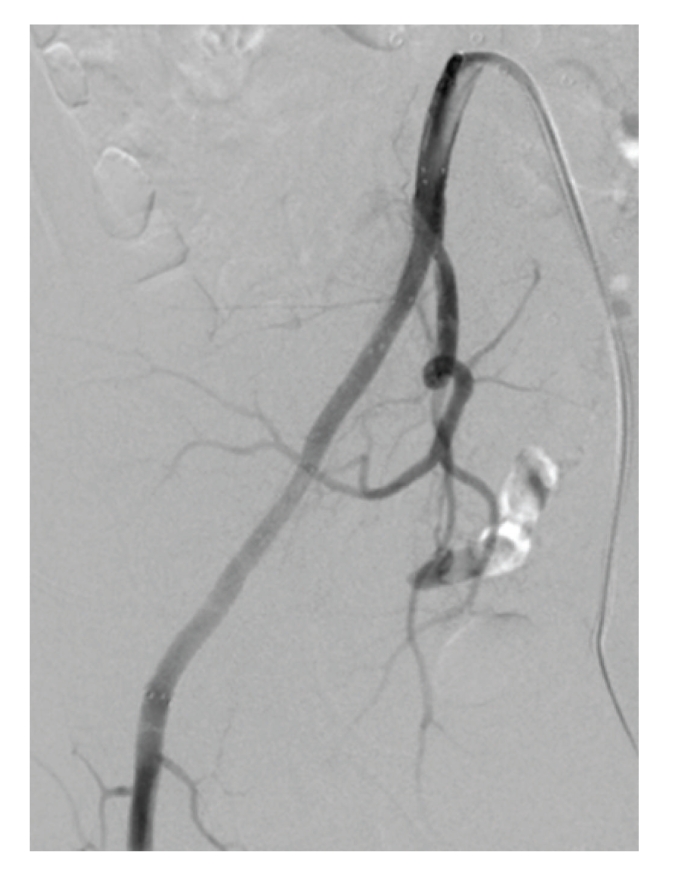
Digital subtraction angiogram, obtained 28 hours after initiation of catheter-directed thrombolysis, shows patency of stented right common and external iliac arteries.

Following eight hours of thrombolytic therapy, a repeat angiogram of the right lower extremity demonstrated near complete lysis of the extensive thrombus. A small amount of residual emboli remained, occluding a branch of the profunda femoris artery and the lateral plantar artery of the foot. Both catheters were exchanged with the catheter tips repositioned to optimize delivery of thrombolytic agent to residual clot. After an additional 12 hours of therapy, angiographic reassessment demonstrated complete lysis of thrombus (Figure 6). The patient remained hemodynamically stable throughout the procedure.

**Figure 7:**
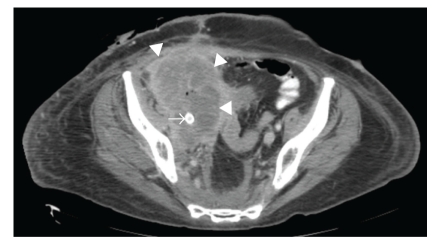
Contrast-enhanced CT scan, obtained four months after-stent insertion, demonstrates interval local tumor progression (arrowheads) but maintained patency of stented iliac artery (arrow).

The patient subsequently clinically improved, with a discharge from the hospital after ten days without any recurrent bleeding episodes or infectious complication of covered stent insertion. Her right lower quadrant abscess drain was uneventfully removed in colorectal surgery clinic approximately two months after initial placement. Follow-up CT scan performed four months after covered stent insertion revealed local progression of right lower quadrant tumor, buttressing the patent stented right external iliac artery (Figure 7). The patient suffered no additional bleeding episodes or infectious events, as evidenced by lack of clinical signs and symptoms as well as hematologic laboratory changes, and subsequently expired due to advanced metastatic cancer involving her liver while in hospice care eight months after-procedure.

## 3. Discussion

The presence of vascular infection significantly complicates conventional surgical and endovascular therapies of pseudoaneurysms. Treatment of infected pseudoaneurysms traditionally involves operative ligation and resection. In a meta-analysis of the literature, M. K. Razavi and M. D. Razavi report an early mortality rate as high as 43% following surgical repair of infected aneurysms depending on patient condition [7], and successful repairs are still associated with amputation rates greater than 20% [8]. With endovascular therapy, infected pseudoaneurysm treatment with covered stent exclusion shows an early mortality rate of 5.6%, and the incidence of late pseudoaneurysm-related mortality and complications are 12.2% and 7.8%, respectively [7]. Similarly, in a meta-analysis of endovascular stent repair for infected aortic aneurysms, Kan et al. reported an overall mortality of 20.8%, with persistent infection in 22.9% of cases [9]. 12-month survival in patients with persistent infection was 39% compared to 94% in the infection-free group [9]. Predictors of adverse outcome in endovascular stent repair of infected aortic aneurysms include aneurysm rupture and the presence of active infection at the time of stent deployment [9]. 

Ordinarily, the use of a covered stent is contraindicated in an area of infection due to potential bacterial colonization of the graft material [9, 10], and covered stent insertion is generally not felt to provide a long-term solution for treatment of infected pseudoaneurysms [8]. While possible alternatives, such as coil embolization with extra-anatomic vascular surgical bypass, were considered in our case, surgery was precluded by poor patient clinical status and expected low likelihood of post-operative survival. The decision to proceed with endovascular pseudoaneurysm exclusion with a covered stent in an infected field was made after considering these factors. Subsequent conventional angiography enabled prompt diagnosis and management, and the patient was treated with an aggressive antibiotic regimen to prevent infection of the newly placed covered stents. Notably, there was no postprocedure laboratory evidence of bacteremia to suggest colonization of the covered stent. 

An additional element of complexity was added to our case when thrombosis developed within the covered stent minutes after deployment, limiting blood flow in the right lower extremity and threatening critical limb ischemia. In general, known active or recent hemorrhage constitute major absolute contraindications for thrombolysis therapy [4, 5]. Additional absolute contraindications include established cerebrovascular events, active bleeding diathesis, recent gastrointestinal bleeding, intracranial or spinal surgery within the last three months, and intracranial trauma [4, 5]. Recent cardiopulmonary resuscitation, recent major nonvascular surgery or trauma, uncontrolled hypertension (systolic blood pressure ≥180 mm Hg or diastolic blood pressure ≥110 mm Hg), intracranial tumor, and puncture of noncompressible vessel constitute relative major contraindications, also increasing hemorrhage risk [4, 5]. Accordingly, use of thrombolysis in our case could have been contraindicated on two grounds: recent bleeding as well as the presence of active infection with possible septic thrombosis. 

On review of the English language literature, the safety of systemic or catheter-directed thrombolysis in the setting of active hemorrhage or other absolute procedure contraindications has not been extensively studied. While sporadic case reports describe the safe use of intravenous [11] or catheter-based [12] thrombolysis in postsurgical patients, for example, large sample size prospective or retrospective investigations assessing the safety thrombolysis in actively bleeding patients have not been performed, likely due to unethical and unnecessary risk to study subjects. While recent major hemorrhage from a bleeding mycotic pseudoaneurysm constituted an absolute contraindication to thrombolysis in our case, we felt that lytic therapy could be pursued safely given the secure treatment of the bleeding source with stent exclusion prior to administration of thrombolytic medications. Although catheter-directed thrombolysis was not the only treatment option, with mechanical thrombectomy, stent-in-stent placement, surgical embolectomy, arterial bypass, and limb amputation as alternatives, it was felt to represent the treatment option with highest likelihood for success and lowest morbidity and mortality rates in our complex and medically unstable patient. Should the patient have developed any signs or symptoms suggesting hemorrhage during the course of therapy, thrombolysis would have been immediately terminated.

## 4. Conclusion

In summary, this case demonstrates successful use of catheter-directed thrombolysis in the setting of recent hemorrhage and an infected field, and therein exemplifies unique application of a traditional catheter-based technique in an unconventional situation. While the use of thrombolysis cannot be advocated in patients with active or recent bleeding events and existing infection on the basis of this single case, the safety of lysis in a nontraditional application is highlighted herein and serves as a reminder to endovascular specialists that measures thought to be contraindicated may rarely be safely performed and can provide benefit to patients, in our case contributing to eight months of prolonged lifespan. As a precaution, however, it warrants mention that the decision to proceed with such an intervention need to be made on a case-by-case basis, with the best interest of the patient taken into account and after thorough discussion of the procedure benefits, risks, and alternatives with the patient, family, and consulting physicians.
